# Spatial analysis of environmental and socioeconomic factors impacting maternal and infant health outcomes in North Carolina

**DOI:** 10.1007/s13412-025-01060-1

**Published:** 2025-11-06

**Authors:** Sarah E. Ulrich, Margaret M. Sugg, Jennifer D. Runkle, Caroline A. Fehlman, Dennis Guignet

**Affiliations:** 1 Department of Geography and Planning, Appalachian State University, P.O. Box 32066, Boone, NC 28608 USA; 2 North Carolina Institute for Climate Studies, North Carolina State University, 151 Patton Avenue, Asheville, NC 28801 USA; 3 Department of Economics, Appalachian State University, P.O. Box 32051, Boone, NC 28608 USA

**Keywords:** Spatial autocorrelation, Cluster detection, Local indicators of spatial autocorrelation, Preterm birth, Low birth weight, Gestational diabetes mellitus, Pregnancy-induced hypertension, Index of Concentration of Extremes, Heatwaves, Extreme heat, Risk-screening environmental indicators

## Abstract

Environmental stressors like high temperatures and pollution, often compounded by socioeconomic factors, have been linked to adverse maternal and birth outcomes. Despite this well-established link, limited studies have examined the spatial relationship between these factors to prioritize community interventions and future research needs. This study examines the spatial trends linking environmental parameters, socioeconomic factors, and adverse maternal and infant health outcomes in North Carolina from 2011 to 2019. Through exploratory spatial data analysis methods, including bivariate Local Indicators of Spatial Association (LISA) and spatial regression, clusters of environmental risks and health outcomes were identified, shedding light on the disproportionate impact of structural racism and income inequality on adverse health outcomes. In addition, this analysis highlights a significant association between hazardous pollutants, as indicated by Risk-Screening Environmental Indicator (RSEI) toxicity-weighted concentrations, and adverse health outcomes, persisting even after adjusting for racial and income segregation. In contrast, heatwaves, defined as any heatwave event across the pregnancy period, showed varied effects and little significance with birth and maternal outcomes. These findings underscore the critical need for targeted interventions addressing socioeconomic disparities and environmental health hazards to enhance maternal and infant health outcomes in North Carolina and similar regions.

**Supplementary Information:**

The online version contains supplementary material available at 10.1007/s13412-025-01060-1.

## Introduction

Cumulative and disproportionate exposure to pollution can adversely affect human health, and these effects may be further exacerbated by exposure to climatic events such as extreme heat. Climate change threatens to increase exposure to differential vulnerability among the most underrepresented populations. This threat has raised a call to action with the World Health Organization, the American College of Obstetricians and Gynecologists, and the Lancet Countdown, identifying human health as one of the greatest consequences of climate change (ACOG 2018; WHO 2018; Watts et al. 2018).

These environmental stressors are occurring against a backdrop of a maternal crisis in the US, with increases in adverse maternal mortality across all racial and ethnic groups (Fleszar et al. 2023). Nevertheless, this increase varies across specific racial groups, with the maternal mortality rate 2.6 times higher for non-Hispanic Black women than non-Hispanic White women (Hoyert 2021). Moreover, the high disparity between non-Hispanic Black and White maternal populations remains stable even after controlling for maternal comorbidities and education (Leonard et al. 2019). These disparities are also pronounced among racial and ethnic minority groups, including American Indians/Alaskan Natives and Hispanic-Americans (Fleszar et al. 2023).

Racial and ethnic minority populations not only face higher rates of maternal mortality but also often live in overburdened and marginalized communities with high exposure to poor air quality and other toxic substances. For instance, previous research has shown that toxic-release inventory sites are located in racial and ethnic minority and low-income communities, and this proximity may impact health outcomes (Charette et al. 2022; Pavan et al. 2023). This disproportionate exposure is well documented in the field of environmental justice research, where communities' disproportionate exposure to environmental hazards is tied to systematic inequalities in environmental regulations and structural elements, including governance, housing, and systemic racism (Alvarez 2023).

Additionally, emerging evidence suggests that everyday environmental exposures, including high ambient temperatures and toxic exposures, such as poor air quality, increase adverse maternal and infant health outcomes (Rylander et al. 2013; Basu et al. 2016). However, limited studies have focused on the impact of climate change and air pollution on women and children in underserved communities in the Southeastern US, despite being a region with an escalating maternal death rate and a high level of health disparities (Singh et al. 2018; Singh 2020; Morello-Frosch and Shenassa 2006; Rylander et al. 2013). This study examines the spatial relationship between environmental parameters (e.g., Risk-Screening Environmental Indicator, Extreme Heat) and birth and maternal outcomes at the census tract level for North Carolina (NC) from 2011 to 2019. Exploratory spatial data analysis techniques are employed to identify clusters or “hot spots” of both environmental and maternal or infant outcomes. Environmental data sources include the risk-screening environmental indicator (RSEI) model, which utilizes toxicity-weighted concentrations, and the National Center for Environmental Information’s nClimdGrid-Daily dataset for temperature at the census tract level. Results identify locations with unusual incidence patterns of adverse maternal health conditions and birth outcomes in relation to environmental factors (e.g., toxicity, heat, economic inequality, and structural racism). The locations of these clusters are priority areas for environmental and health intervention.

## Data

### Study area

The study is conducted with census tract-level data for North Carolina, a state situated in the southeastern region of the United States (Supplemental Fig. 1).

### Maternal and birth outcomes

The outcomes for this study were obtained from the Vital Statistics Department of the North Carolina State Center for Health Statistics, and research was conducted under an agreement with the Children’s Environmental Health Initiative (CEHI). Data includes detailed birth records for all live births in NC, including information on maternal demographics, maternal and infant health, and maternal obstetrics history. Location was identified using the mother’s place of residence at the time of birth to link birth records to census tract environmental and sociodemographic data. Four outcomes were analyzed in this analysis: preterm birth (PTB), low birth weight (LBW), gestational diabetes mellitus (GDM), and pregnancy-induced hypertension (PIH). LBW was considered a live birth under 2500 g, and PTB as a live birth under 37 weeks of gestation.

### Risk Screening Environmental Indicators (RSEI) model

Ambient concentrations of hazardous air pollutants (HAPS) were obtained from the US EPA’s the Risk Screening Environmental Indicators Geographic Microdata (RSEI), a dataset covering the contiguous United States with 810 m2 grid cells. Each grid cell contains modeled estimates of the annual average ambient air concentration values for numerous HAPs. The aggregated RSEI data incorporate two fundamental variables: a risk-related score and a toxicity-weighted average ambient concentration. The RSEI data report spatially explicit, annual values for these variables. The annual values are modelled based on the quantity and type of chemical emissions reported by individual firms to the US EPA’s Toxic Release Inventory (TRI). The reported emission quantities are input into the American Meteorological Society/EPA Regulatory Model (AERMOD), which accounts for wind speed and direction, ambient temperature, and observed cloud cover to determine chemical transport and final ground-level air concentrations (United States Environmental Protection Agency 2023). The annual ambient air concentrations for each grid cell are based on the HAP emissions reported to the TRI by all facilities within a 50-mile radius, and for all reported chemicals. Toxicity-weighted average annual ambient concentrations are calculated by the EPA across all reported chemicals, thus providing a comprehensive and spatially detailed measure of HAPs, accounting for numerous chemicals and across many polluting facilities, including those in neighboring census tracts. Aggregated RSEI microdata for core chemicals were obtained at the 2010 census tract level from the EPA (United States Environmental Protection Agency 2023).

### Heat wave exposure

Average daily temperature data were collected at the census tract level for the contiguous United States using the National Center for Environmental Information’s nClimdGrid-Daily from 2011 to 2019. Data were derived from observations from the Global Historical Climatology Network daily dataset, with additional processing for spatial and temporal variations (Durre et al. 2022). Heatwaves were defined as three consecutive days with temperatures above a 95% threshold (Shiva et al. 2019; Brooke Anderson and Bell 2011). Sensitivity analyses were performed using definitions of two- and four-day durations and a 90% and 99% threshold (Supplemental Figs. 2 and 3). Temperature thresholds were defined at the climate division level to account for local differences in acclimatization. North Carolina’s eight climate divisions are calculated by NOAA using area-weighted daily temperature and precipitation values (Karl and Koss 1984).

### Built environment

The neighborhood was defined at the census tract level, and community-level contextual variables were derived for this analysis, including structural racism and economic residential segregation, as measured through the Index of Concentration at the Extremes (ICE) (Chambers et al. 2020; Hardeman et al. 2022). Originating from Massey's work in 2001, the Index of Concentration at the Extremes (ICE) measures the concentration of a population into relative extremes of advantage and deprivation. Building on this framework, Krieger et al. extended ICE's application to race and income, introducing ICEIncome and ICERace. This approach offers several advantages, encompassing spatial and social polarization simultaneously, thereby addressing common multi-collinearity issues associated with separate measures of advantage and deprivation. ICE variables were derived from the 2010 Census, and a score of − 1 indicates that 100% of the population within a tract was the most deprived (i.e., low-income or majority Black and non-white Hispanic), while a score of 1 indicates that 100% of the population was the most privileged (i.e., high-income or majority white). The primary ICE Race metric assessing structural racism was calculated using non-Hispanic white individuals as the ‘privileged’ group and the combined population of non-Hispanic Black and non-white Hispanic individuals as the ‘deprived’ group. To assess the sensitivity of our findings to different ICE Race definitions, ICE Race was also calculated using (1) non-Hispanic white vs. Hispanic and (2) non-Hispanic white vs. non-Hispanic Black..

## Methods

### Exploratory spatial analysis

An exploratory spatial data analysis approach was adopted to study toxicity patterns, heatwaves, and maternal and birth outcomes. This technique cannot explain causal mechanisms, but it allows for generating new hypotheses instead of validating them a priori (Nelson and Brewer 2017). Univariate local indicators of spatial autocorrelation (LISA) and bivariate LISA statistics were calculated. Unlike global techniques of spatial autocorrelation, LISA allows for the specification of clusters. Univariate LISA assesses spatial autocorrelation at the census-tract level for a single variable using the Local Moran’s *I* statistic, an extension of the global Moran’s* I* index. The bivariate LISA extends the application of Local Moran’s *I* to identify the presence or absence of significant spatial clusters or outliers across two variables. Identifying outliers may indicate an unknown localized process that uniquely produces disadvantages or advantages.The Queen contiguity matrix was used to define neighbors, which considers all census tracts with a common boundary as neighbors. The following conditions: 1) PTB, 2) LBW, 3) PIH, and 4) GDM, were evaluated across all environmental factors (e.g., built environment, toxicity, heat). Each condition was calculated as a rate, normalized by the total number of births within each census tract.. Hot spots, cold spots, and spatial outliers that emerged in this analysis are defined as:

- **Hot Spots**: Positive spatial autocorrelation or locations with high values, with similar neighbors (High-High).
- **Cold Spots**: Positive spatial autocorrelation or locations with low values, with similar neighbors (Low-Low).
- **Spatial Outliers**: Negative spatial autocorrelation or locations with high values but low-value neighbors (High-Low) and locations with low values but with higher neighbors' values (Low–High).

Tracts not found to have a significant spatial association with their neighboring tracts are deemed insignificant. The bivariate LISA does not account for in-situ correlation. Global spatial autocorrelation statistics were also reported by averaging local values (Anselin 2005). Statistical significance was considered at alpha = 0.05. LISA analyses were performed in the GeoDa software package version 1.22.0.4 (Anselin 1995; Anselin et al. 2006). ArcGIS Pro version 3.3.1 was used for mapping.

### Spatial regression

A spatial lag model was used to account for spatial autocorrelation among the observed variables. The inclusion of a spatially lagged dependent variable in the regression equation captures the potential influence of neighboring observations from census tracts. This approach enables the modeling of spatial interdependence among observations, acknowledging that the value of a given variable at a particular location may be influenced by the values of the same variable at nearby locations (Anselin and Bera 1998). A contiguity-based queen’s spatial weight matrix was used to define the spatial relationships among observations, and the specification of the spatial lag model was determined by comparing the robust Lagrange multiplier (LM) tests for the spatial autoregressive errors and the spatial lag (Florax et al. 2003). Regression analysis was performed in RStudio version 2025.05.0 using the ‘spdep’ package and the ‘spatialreg’ package and R software version 4.5.1.

## Results

PTB and LBW were the most common conditions among pregnant persons (8.3%, *n* = 83,270, and 7.3%, *n* = 73,528). PIH and GDM were less frequent than PTB and LBW, with rates of 6.4% (*n* = 64,665) and 5.8% (*n* = 57,821), respectively (Table 1).

**Table 1:** Total case counts and demographic variables for preterm birth (PTB), low birth weight (LBW), gestational diabetes mellitus (GDM), and pregnancy-induced hypertension (PIH) cases

Dimension	Indicator	PTB	LBW	GDM	PIH	Overall
Case Count	n	83,270 (8.3)	73,528 (7.3)	57,821 (5.8)	64,665 (6.4)	1,003,543 (100.0)
PTB (%)	No	0	26,579 (36.1)	51,456 (89.0)	51,461 (79.6)	920,273 (91.7)
	Yes	83,270 (100.0)	46,949 (63.9)	6365 (11.0)	13,204 (20.4)	83,270 (8.3)
LBW (%)	No	36,321 (43.6)	73,528 (100.0)	53,571 (92.6)	52,850 (81.7)	930,015 (92.7)
	Yes	46,949 (56.4)	0	4250 (7.4)	11,815 (18.3)	73,528 (7.3)
GDM (%)	No	76,905 (92.4)	69,278 (94.2)	57,821 (100.0)	57,267 (88.6)	945,722 (94.2)
	Yes	6365 (7.6)	4250 (5.8)	0	7398 (11.4)	57,821 (5.8)
PIH (%)	No	70,066 (84.1)	61,713 (83.9)	50,423 (87.2)	64,665 (100.0)	938,878 (93.6)
	Yes	13,204 (15.9)	11,815 (16.1)	7398 (12.8)	0	64,665 (6.4)
Race/Ethnicity (%)	White	29,578 (35.5)	21,999 (29.9)	22,440 (38.8)	29,097 (45.0)	437,094 (43.6)
	Black	15,139 (18.2)	15,732 (21.4)	7926 (13.7)	10,922 (16.9)	139,247 (13.9)
	Hispanic	13,907 (16.7)	11,069 (15.1)	13,643 (23.6)	9266 (14.3)	182,924 (18.2)
	Other/Unknown	24,646 (29.6)	24,728 (33.6)	13,812 (23.8)	15,380 (23.8)	244,278 (24.4)
	Unknown	180 (0.2)	173 (0.2)	81 (0.1)	119 (0.2)	1757 (0.2)
Mother's Age (%)	15–19	6290 (7.6)	6564 (8.9)	1561 (2.7)	4601 (7.1)	68,474 (6.8)
	20–24	19,975 (24.0)	19,388 (26.4)	8590 (14.9)	15,425 (23.9)	237,951 (23.7)
	25–29	22,644 (27.2)	19,833 (27.0)	15,269 (26.4)	18,298 (28.3)	292,235 (29.1)
	30–34	20,060 (24.1)	16,489 (22.4)	17,812 (30.8)	16,029 (24.8)	258,431 (25.8)
	35–39	11,328 (13.6)	8842 (12.0)	11,511 (19.9)	8309 (12.8)	121,518 (12.1)
	40–44	2973 (3.6)	2412 (3.3)	3078 (5.3)	2003 (3.1)	24,934 (2.5)
WIC (%)	Yes	39,972 (48.1)	38,467 (52.5)	28,914 (50.1)	29,824 (46.2)	453,526 (45.3)
	No	43,077 (51.9)	34,851 (47.5)	28,789 (49.9)	34,722 (53.8)	548,142 (54.7)
Insurance Type (%)	Medicaid	42,609 (51.2)	41,674 (56.7)	25,756 (44.5)	29,071 (45.0)	436,891 (43.6)
	Private	31,242 (37.5)	24,414 (33.2)	24,127 (41.7)	29,551 (45.7)	446,771 (44.6)
	Self-Pay	6182 (7.4)	4918 (6.7)	5809 (10.0)	3527 (5.5)	72,033 (7.2)
	Other	3210 (3.9)	2501 (3.4)	2122 (3.7)	2514 (3.9)	46,933 (4.7)
Low Income (%)	No	31,375 (37.7)	24,064 (32.7)	23,608 (40.8)	28,220 (43.6)	447,492 (44.6)
	Yes	51,895 (62.3)	49,464 (67.3)	34,213 (59.2)	36,445 (56.4)	556,051 (55.4)

### Spatial analysis

#### Univariate LISA analysis

Univariate Moran’s *I* statistics for all maternal and birth outcomes (PTB, LBW, GDM, and PIH) are shown in Supplemental Table 1. The global Moran’s *I* for all birth and maternal health outcomes indicates a high positive spatial autocorrelation, with LBW being the most clustered (Moran *I:* 0.443), followed by PTB (Moran *I:* 0.297), GDM (Moran *I:* 0.284), and PIH (Moran *I:* 0.268) (Supplemental Table 1). All of Moran’s *I* values were significant at *p* < 0.05.

**Fig. 1:**
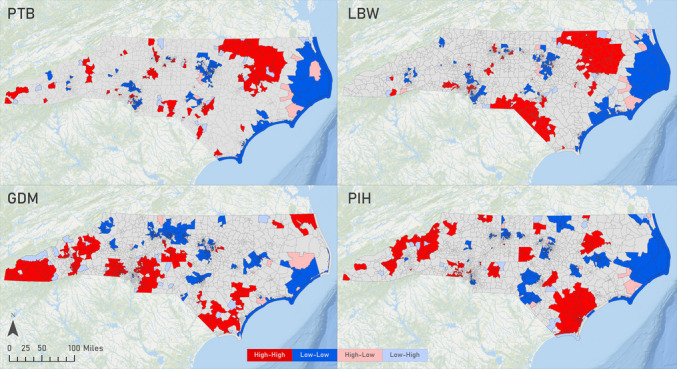
Univariate LISA clusters calculated for each birth and maternal outcome

Univariate LISA clusters were calculated for each birth and maternal outcome and are mapped in Fig. 1. High-high clusters are hotspot areas with a greater prevalence of each birth or maternal outcome. Hotspots for PTB and LBW are in northeastern North Carolina. GDM and PIH exhibited a different pattern, with more hot spots in the western part of North Carolina.

The selected demographic variables for women residing in census tracts identified as high-high clusters for each outcome are shown in Table 2. Table 3 lists additional census variables for tracts identified in high-high clusters. Substantial disparities exist for indicators of race for PTB and LBW, and income disparities exist for all outcomes. Similar neighborhood-level disparities are observed among birth and maternal outcome hotspots in relation to tract-level socioeconomic indicators and all other census tracts in North Carolina. Maternal outcome (GDM, PIH) hotspots differ in that they are composed of a greater proportion of white women. They are located in areas with lower incomes and less diversity than other areas of the state. Birth outcomes (PTB, LBW) clusters are defined by a greater proportion of Black women and are located in areas with higher concentrations of racial and ethnic minority populations.

**Table 2:** Demographic composition of women residing in univariate high-high (HH) clusters for preterm birth (PTB), low birth weight (LBW), gestational diabetes mellitus (GDM), and pregnancy-induced hypertension (PIH) (2011–2019)

Dimension	Indicator	PTB HH Clusters	LBW HH Clusters	GDM HH Clusters	PIH HH Clusters	All Births
n	# of births	108,531	139,449	151,637	94,400	1,003,543
Age	35 to 44 (%)	10.3	10.2	12.2	12.2	14.6
Income	WIC (%)	64.6	65.7	51.4	52.2	45.4
Income	Medicaid (%)	66.2	66.9	52.2	52.4	43.5
Race/Ethnicity	White (%)	22.8	16.8	44.8	50.2	43.6
Race/Ethnicity	Black (%)	26.9	27.5	11.6	11.6	13.9
Race/Ethnicity	Hispanic (%)	16.7	17.2	19.6	14.8	18.2

**Table 3:** Select census variables for tracts identified in univariate high-high clusters for preterm birth (PTB), low birth weight (LBW), gestational diabetes mellitus (GDM), and pregnancy-induced hypertension (PIH) using data from the 2010 ACS with 5-year estimates

Dimension	Indicator	PTB HH Clusters	LBW HH Clusters	GDM HH Clusters	PIH HH Clusters	All tracts
n	# of census tracts	242	301	345	232	2195
Education	No high school (%)	24.18	25.90	20.27	19.37	16.38
Employment	Unemployment (%)	13.04	13.76	9.58	9.56	9.04
Housing	Occupied housing units: Renters (%)	44.61	46.55	32.08	30.79	33.82
Housing	Mobile homes (%)	13.63	14.75	17.45	20.35	13.55
Housing	Median housing value for owner-occupied housing units ($)	103,581	96,151	135,471	143,149	166,613
Housing	Median gross rent ($)	633	636	692	688	742
Income	Median Household Income ($)	$33,343	31,880	41,925	41,568	48,952
Dependence	Households receiving social security benefits (%)	31.97	31.61	30.87	33.39	28.03
Mobility	Total Population: No Vehicle Available (%)	13.66	14.41	6.72	7.51	6.90

#### Bivariate LISA analysis

Due to the exploratory nature of LISA, the resulting spatial clusters lack statistical explanatory power. However, they indicate places to focus further attention (Anselin et al. 2007). The number of census tracts identified in high-high (HH), low-low (LL), high-low (HL), and low–high (LH) clusters for heatwaves, toxicity, ICE Race, ICE Income with birth (PTB, LBW), and maternal (GDM, PIH) outcomes are displayed in Table 4.

**Table 4:** Total number of census tracts identified in High-High (HH), High-Low (HL), Low-Low (LL), and Low–High (LH) bivariate local indicators of spatial autocorrelation (LISA) clusters for environmental stressors (e.g., Heatwave, TOxicity, ICE Race, ICE Income) and preterm birth (PTB), low birth weight (LBW), gestational diabetes mellitus (GDM), and pregnancy-induced hypertension (PIH). High-low (HL) clusters are areas where high values of a stressor are surrounded by low values of an outcome, while Low–high (LH) clusters are areas where low stressor values are surrounded by high outcome values

Stressor	Health Outcome	High-High	Low-Low	High-Low	Low–High	Not significant
Heatwave	PTB	176	92	128	247	1552
	LBW	212	122	126	267	1468
	PIH	128	118	136	248	1565
	GDM	226	108	166	311	1384
Toxicity	PTB	108	290	196	49	1552
	LBW	119	329	219	60	1468
	PIH	70	302	194	64	1565
	GDM	79	337	313	82	1384
ICE Race	PTB	76	79	228	260	1552
	LBW	56	77	282	312	1468
	PIH	189	161	75	205	1565
	GDM	263	190	129	229	1384
ICE Income	PTB	52	47	252	292	1552
	LBW	41	67	297	322	1468
	PIH	79	139	185	227	1565
	GDM	130	177	262	242	1384

All clusters are significant at the p < 0.05 level

#### Extreme heat

**Fig. 2:**
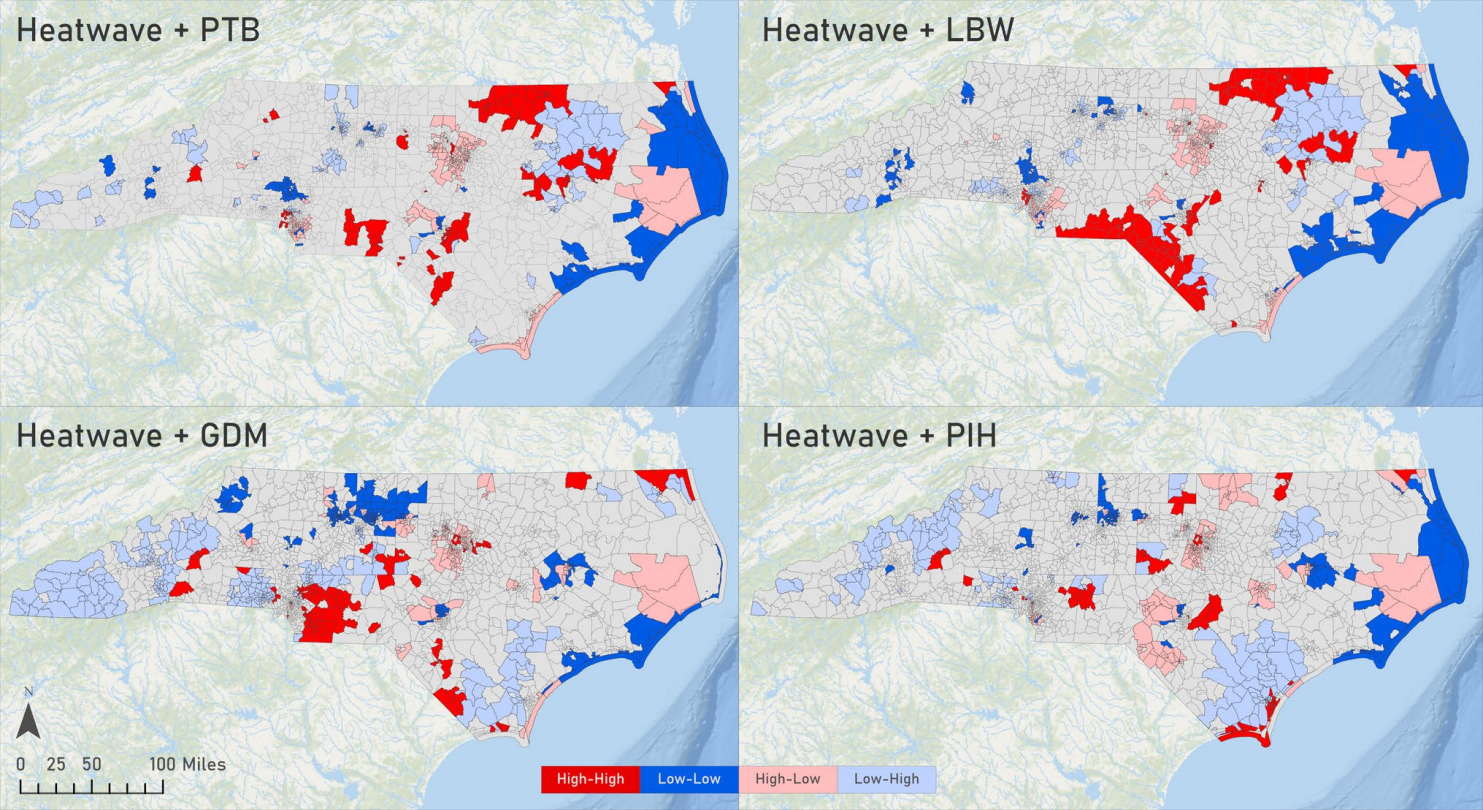
Bivariate LISA maps revealing clusters of positive and negative spatial autocorrelation for heatwaves and birth and maternal outcomes

The bivariate Moran’s *I* for heatwaves and LBW, PTB, PIH, and GDM are shown in Supplemental Table 2. Results for GDM and PIH indicate low negative spatial autocorrelation, suggesting that areas with high numbers of heatwave events are surrounded by areas with low numbers of adverse maternal outcomes (i.e., GDM and PIH). PTB also indicates low negative spatial autocorrelation (*I* = -0.031). LBW indicates low positive (e.g., high-high and low-low) spatial autocorrelation, where census tracts with higher than average heatwave events are surrounded by tracts with higher rates of LBW. Bivariate LISA maps reveal clusters of positive and negative spatial autocorrelation for heatwaves and birth and maternal outcomes (Fig. 2), showing distinct geographic variation in the distribution of heatwave exposure and health outcomes across the state. All cluster locations are significant at the p < 0.05 threshold. GDM shows the greatest number of census tracts in high-high clusters with heatwaves (226), while PIH shows the lowest (128) (Table 4).

#### Toxicity

**Fig. 3:**
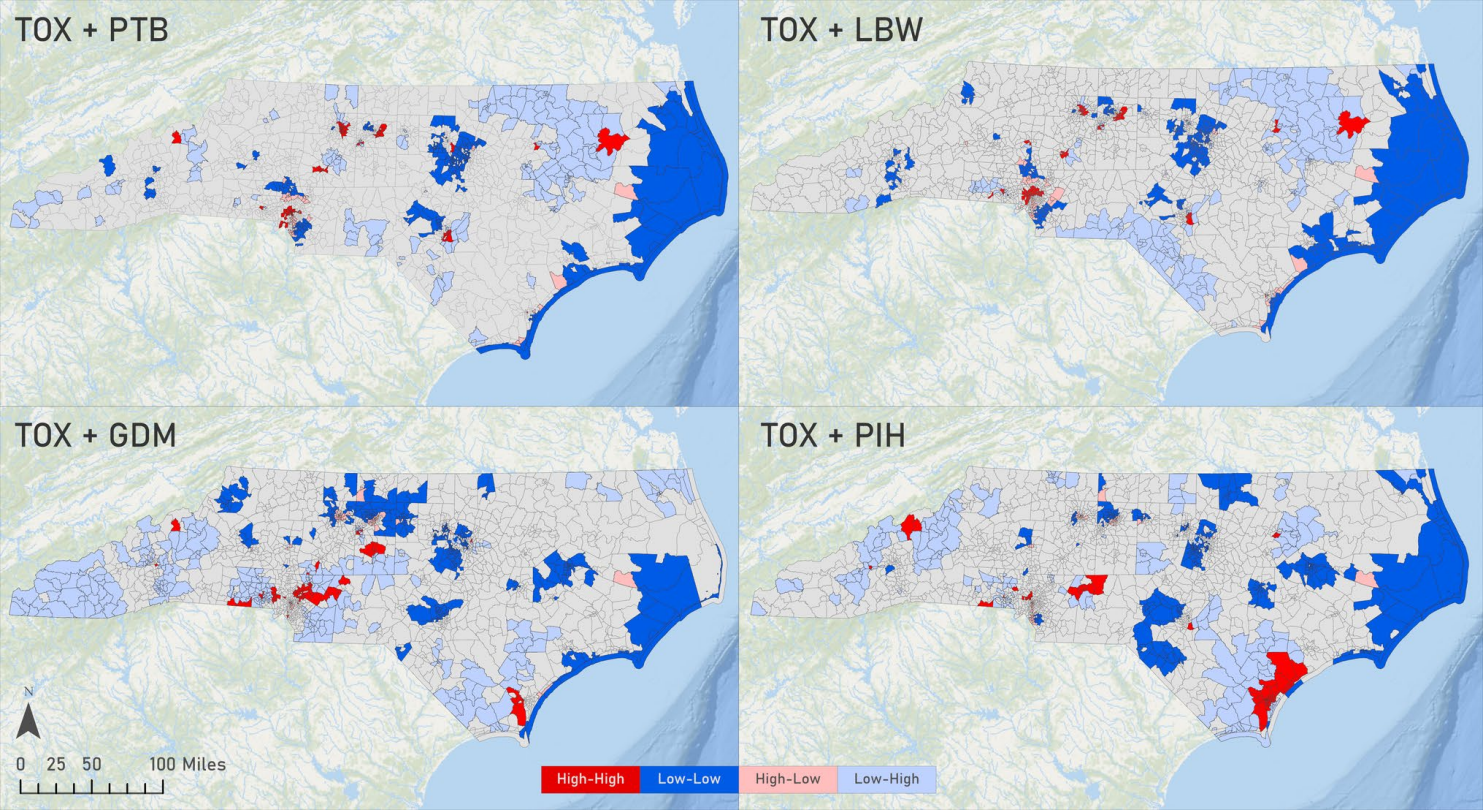
Bivariate LISA maps for toxicity and birth and maternal outcome clusters

The bivariate Moran’s *I* for toxins and all maternal and birth outcomes (PTB, LBW, GDM, and PIH) are shown in Supplemental Table 2. Results for maternal outcomes (GDM and PIH) indicate low negative spatial autocorrelation (e.g., high-low and low–high) for most locations, denoting locations of bivariate extremes or spatial outliers. Results for PTB and LBW indicate low positive (e.g., high-high and low-low) spatial autocorrelation. Bivariate LISA maps for toxicity and birth and maternal outcome clusters are shown in Fig. 3. Bivariate LISA identified a greater number of census tracts in high-high clusters for toxicity and birth outcomes (PTB: 108, LBW: 119) compared to maternal outcomes (PIH: 70, GDM: 79) (Table 4).

#### ICE

**Fig. 4:**
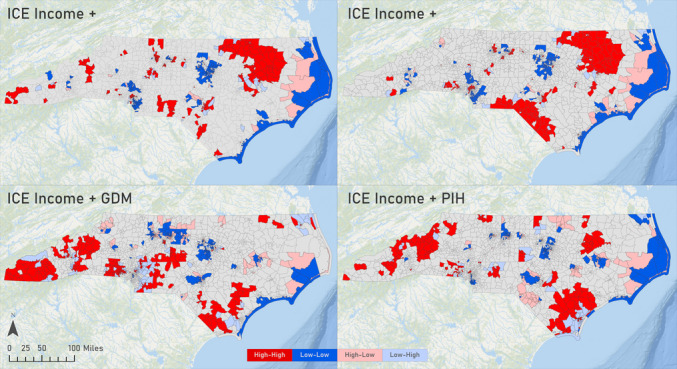
Bivariate LISA maps for ICE Income and birth and maternal outcome clusters

The bivariate Moran’s *I* for ICE Income and all maternal and birth outcomes (PTB, LBW, GDM, and PIH) are shown in Supplemental Table 2. Results for all outcomes indicate negative spatial autocorrelation, suggesting that areas with low levels of adverse birth and maternal outcomes surround locations with high levels of economic privilege. Bivariate LISA maps for ICE Income and birth and maternal outcome clusters are shown in Fig. 4.

**Fig. 5:**
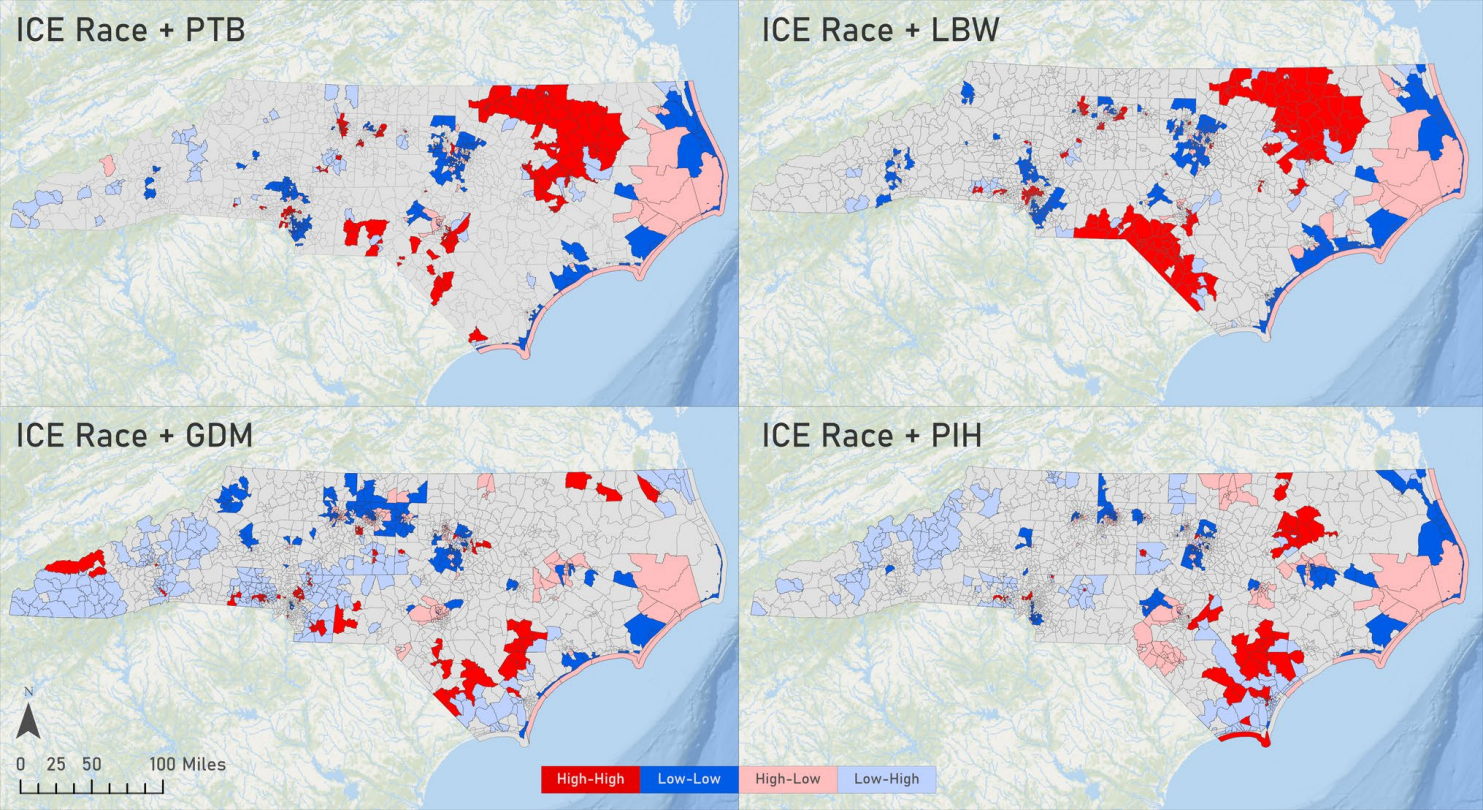
Bivariate LISA clusters for ICE Race and birth and maternal outcomes

The bivariate Moran’s I for ICE Race and birth outcomes (PTB and LBW) indicates negative spatial autocorrelation. In contrast, the bivariate Moran’s I for ICE Race and maternal outcomes (GDM and PIH) indicate positive spatial autocorrelation, demonstrating that areas with high levels of racial segregation are surrounded by areas with high levels of adverse maternal health outcomes and low levels of adverse birth outcomes (Fig. 5). Bivariate LISA clusters for ICE Race and birth and maternal outcomes are shown in Fig. 5.

Maternal outcomes displayed more high-high clustering with ICE outcomes than birth outcomes. A greater number of census tracts within high-high clusters were identified for ICE Race and maternal outcomes (PIH: 189, GDM: 263) compared to birth outcomes (PTB: 76, LBW: 56) (Table 4). A greater number of census tracts in high-high clusters was also identified for ICE Income and maternal outcomes (PIH: 189, GDM: 263) compared to birth outcomes (PTB: 76, LBW: 56) (Table 4).

### Regression

LM tests of the classic ordinary least squares (OLS) regression were used to determine the fixed effects and the type of spatial dependence (error vs. lag). Results indicated that the LM lag was the most robust indicator of spatial dependence. The model fit improved compared to the OLS model, which indicated lower AIC values.

Table 5 summarizes the OLS and the spatial regression results for each of the four outcomes (GDM, PIH, LBW, PTB). Environmental and community-level independent variables included Toxicity, Heatwaves, Structural Racism (ICE Race), Income Inequality (ICE Income), and rural–urban classification (Urban, Micropolitan, Small Town, and Rural/Isolated). The spatial lag regression coefficients for Toxicity, Heatwaves, Rural, Micropolitan, and Small Town residence were insignificant, except for a slight negative correlation between PIH and Toxicity (-0.001) and micropolitan residence (-0.003).

**Table 5:** Spatial Regression and Ordinary Least Square Regression Results for Gestational Diabetes (GDM), Pregnancy Induced Hypertension (PIH), Low Birth Weight (LBW), and Preterm Birth (PTB)

	GDM		PIH		LBW		PTB	
	OLS	Spatial Lag	OLS	Spatial Lag	OLS	Spatial Lag	OLS	Spatial Lag
(Intercept)	0.053***	0.015***	0.060***	0.020***	0.074***	0.047***	0.082***	0.052***
	(0.001)	(0.001)	(0.001)	(0.001)	(0.001)	(0.002)	(0.001)	(0.002)
Toxicity	− 0.001	− 0.000	− 0.001*	− 0.001*	0.000	0.000	0.001	0.000
	(0.000)	(0.000)	(0.001)	(0.000)	(0.000)	(0.000)	(0.000)	(0.000)
Heatwaves	− 0.002***	− 0.000	− 0.002**	− 0.000	− 0.000	− 0.000	− 0.001*	− 0.000
	(0.001)	(0.000)	(0.001)	(0.000)	(0.000)	(0.000)	(0.000)	(0.000)
Structural Racism	− 0.005***	− 0.001	− 0.007***	− 0.002*	0.019***	0.014***	0.010***	0.008***
	(0.001)	(0.001)	(0.001)	(0.001)	(0.001)	(0.001)	(0.001)	(0.001)
Income Inequality	0.020***	0.010***	0.026***	0.013***	0.043***	0.033***	0.039***	0.029***
	(0.002)	(0.002)	(0.002)	(0.002)	(0.002)	(0.002)	(0.002)	(0.002)
Urban	Reference		Reference		Reference		Reference	
Micropolitan	0.002	0.000	− 0.004*	− 0.003*	0.001	− 0.001	0.001	− 0.001
	(0.001)	(0.001)	(0.001)	(0.001)	(0.001)	(0.001)	(0.001)	(0.001)
Rural	0.011***	0.003	0.003	− 0.001	− 0.002	− 0.003	0.001	− 0.001
	(0.003)	(0.002)	(0.003)	(0.002)	(0.002)	(0.002)	(0.002)	(0.002)
Small Town	0.005*	0.000	0.002	− 0.001	0.001	0.000	0.002	0.001
	(0.002)	(0.002)	(0.002)	(0.002)	(0.002)	(0.002)	(0.002)	(0.002)
rho		0.700***		0.660***		0.374***		0.373***
		(-0.020)		(-0.021)		(-0.025)		(-0.027)
Num.Obs	2165	2165	2165	2165	2165	2165	2165	2165
R2	0.070		0.074		0.460		0.295	
R2 Adj	0.067		0.071		0.458		0.292	
AIC	− 10,167.0	− 11,137.6	− 10,084.0	− 10,899.4	− 10,890.5	− 11,101.3	− 10,631.0	− 10,807.8

^***^ *p* < 0.001; ** *p* < 0.01; * p < 0.05

Regression results for structural racism (ICE Race [Hispanic + Black vs. non-Hispanic white]) identified a positive association with birth outcomes (LBW: 0.014, PTB: 0.008)) and a negative association with PIH (-0.002). There is a strong positive association between income inequality (ICE Income) and all outcomes (*GDM; 0.010, PIH: 0.013, LBW: 0.033, PTB: 0.029). Sensitivity analyses of alternative structural racism metrics (Non-Hispanic white vs. Black and Non-Hispanic white vs. Hispanic) are included in Supplemental Tables 3 and 4.

The spatial autoregressive coefficient (Rho) captures the extent to which outcomes in one census tract are influenced by neighboring census tracts. The resulting models for maternal and birth outcomes have positive and statistically significant values of Rho, indicating that there is meaningful spatial dependence in the data. The strongest effects were observed for maternal outcomes (GDM: Rho: 0.70, PIH: Rho: 0.66).

## Discussion

This analysis investigates the spatial association between maternal and birth outcomes and environmental parameters, such as RSEItoxicity-weighted concentrations, extreme heat, as well as income and racial segregation measures in North Carolina from 2011 to 2019. The study identifies clusters or "hot spots" of environmental risks and adverse maternal or infant health outcomes by employing bivariate LISA and spatial regression as exploratory spatial data analysis techniques. The results highlight priority populations for environmental health intervention by pinpointing locations where adverse maternal health conditions and birth outcomes exhibit unusual incidence patterns. In general, strong trends are observed between measures of structural racism and income inequality, predicting spatial patterns of adverse maternal (e.g., pregnancy-induced hypertension and gestational diabetes) and infant outcomes (e.g., low birth weight and preterm birth).

This work noted different spatial trends between maternal outcomes (e.g., PIH and GDM) and birth outcomes (e.g., LBW, PTB). Hotspots of adverse infant outcomes were concentrated in the northeastern part of the state, a region with more racial and ethnic minority populations. The spatial regression confirms this finding, with racial segregation predicting higher rates of LBW and PTB compared to PIH and GDM. A high positive Moran’s *I* value for LBW suggests that this outcome is more likely to concentrate in specific vulnerable subpopulations. In addition, the results further indicate that PTB not only disproportionately affects racial minorities and socio-economically disadvantaged women but also communities primarily comprised of them (Schaaf et al. 2013; McHale et al. 2022). Although this finding has been well-documented for the association between income inequality and PTB (Huynh et al. 2005, 2018; Vos et al. 2014), more research is needed to establish an association between birth outcomes and racial inequality measures.

Self-reported experiences of racial discrimination from pregnant and early-postpartum Black persons were significantly higher in neighborhoods with both income and racial segregation in Oakland, California, also measured through the Index Concentration of Extremes (Chambers et al. 2020). These findings suggest similar experiences in North Carolina; future research is needed to confirm similar patterns at the individual level. Eliminating disparities between birth outcomes is of the utmost importance as these conditions strongly predict infant mortality and neurodevelopmental outcomes in children and are associated with chronic disease and lower life expectancy in adulthood (MacDorman 2011; Swamy and SkjŠrven 2008; Waitzman et al. 2021, Frey and Klebanoff 2016).

Cluster locations for maternal outcomes (GDM and PIH) were more prominent in the western part of North Carolina, as well as in the southern Piedmont and southeastern coastal regions. These are areas where populations tend to have lower incomes and less diversity than in other areas of the state. The findings of higher rates of pregnancy-induced hypertension and gestational diabetes in predominantly white communities contrast with the literature, which finds that non-Hispanic Black populations have a higher prevalence of PIH and GDM than non-Hispanic populations (Singh et al. 2018; Dabelea et al. 2005). Specific maternal outcomes like PIH have an established relationship with socioeconomic status (Heshmati et al. 2013), and this work highlights that this relationship extends to neighborhood-level measures of income inequality. Future analysis is needed at an individual level to understand if individual risk factors alter these findings.

The effects of RSEI toxicity-weighted concentrations on birth or maternal outcomes were insignificant or mixed.While growing evidence supports associations between air pollutant exposure during pregnancy and adverse outcomes (Hu et al. 2014; Pedersen et al. 2014; Bai et al. 2020), research specifically examining HAPs and maternal health remains limited. Previous studies have found associations between HAP exposure and newborn health (Currie and Schmieder 2009; Currie et al. 2011), with some evidence linking specific HAPs to hypertensive pregnancy disorders (Walker et al. 2022; Zhu et al. 2017). Our null findings may reflect the complex nature of cumulative toxicity exposure captured by RSEI, which aggregates multiple pollutants across sources. A limited number of high-high clusters were identified in the central Piedmont region for all outcomes, and in the southeastern coastal region for maternal outcomes. The distinct spatial patterns that emerged with PTB, PIH, GDM, and LBW consisted of low–high clusters throughout NC and low-low clusters along the coast for all outcomes. RSEI toxicity-weighted concentrations are primarily found in urban locations (Pavan et al. 2023) and are higher in racial and ethnic minority neighborhoods.

Surprisingly, the frequency of heatwaves had an insignificant or mixed effect across birth and maternal outcomes. This unexpected finding may be attributed to our methodological approach of aggregating heatwave exposure across the entire study period (2011–2019), which included both unusually warm years (2016, 2017, 2019) and cooler years (2013, 2014). This temporal aggregation may have masked the acute effects of extreme heat events that occur in specific years. Additionally, Locations with the highest frequency of heatwaves experienced lower rates of birth outcomes, and no impact on maternal outcomes. The effect of heatwaves is less notable than income and racial segregation; future analyses should use more robust environmental epidemiological methods to tease out this association, such as distributed lag-nonlinear models (Gasparrini 2022). In addition, the operationalization of heatwaves was specific to select studies (Shiva et al., 2019; Brooke Anderson and Bell 2011) and could be refined to account for local climatology and acclimatization of the underlying population.

### Strengths and Limitations

This study had several notable strengths. First, the use of an exploratory spatial analysis approach allowed for the identification of community-level drivers associated with maternal and birth outcomes and locations that experience an abnormally high burden of these outcomes. This approach enables the identification of clusters or "hot spots" of environmental risks and adverse health outcomes, providing valuable insights for targeted intervention strategies and resource allocation. The inclusion of a spatial effect in the spatial regression models enables consideration of how census tract-level case counts are influenced by case counts in neighboring areas, while also assessing the impact of community-level factors that contribute to higher levels of maternal and birth outcomes.

There are also some notable limitations in this study. First, the study is based on vital records, which are typically recorded after delivery at the birth hospital. The data are, therefore, limited to those fields mandated by the vital records collection process, some of which are self-reported. A key limitation is that individual-level characteristics could not be accounted for, which may explain some disparities. However, previous work has shown that even accounting for individual-level factors did not fully explain differences between outcomes like PTB and community-level income inequality (Huynh et al. 2005). Second, the temporal resolution of our environmental data presents limitations. RSEI data is only available annually, preventing examination of seasonal variations in toxicity exposure. Similarly, our approach of aggregating all years (2011–2019) may have obscured important temporal patterns, particularly for heat exposure, where year-to-year variations were substantial. Future research employing time-stratified analyses could better capture these temporal dynamics. Third, as an ecological study design, our findings are subject to ecological fallacy—associations observed at the census tract level may not reflect individual-level relationships. While our spatial approach identifies priority areas for intervention, individual-level studies are needed to confirm these associations. Lastly, our ecological study design treats each birth as an independent observation and does not account for multiple births from the same individual over the study period. This may lead to overrepresentation of women with multiple pregnancies in the spatial patterns, particularly if they remained in the same census tract throughout the study period.

## Conclusion

This analysis investigated the spatial relationship between environmental parameters, socioeconomic factors, and adverse maternal and infant health outcomes in North Carolina from 2011 to 2019. Employing exploratory spatial data analysis techniques, the study identified clusters of environmental risks and health outcomes, highlighting the disproportionate impact of structural racism and income inequality on adverse health outcomes. While the frequency of heatwaves and high environmental toxicity showed mixed effects, income and racial segregation emerged as strong predictors of adverse health outcomes. These results underscore the urgent need for targeted interventions to address socioeconomic disparities and environmental health hazards, aiming to improve maternal and infant health outcomes in North Carolina and similar regions.

## Supplementary Information

Below is the link to the electronic supplementary material.

Supplementary file1

Supplementary file2

Supplementary file3

Supplementary file4

## Data Availability

Data used in this study are protected health information and are not available for public use or access.
