# Supplementary material for: Spatial analysis of environmental and socioeconomic factors impacting maternal and infant health outcomes in North Carolina
Source: J Environ Stud Sci. 2025 Nov 6;16(3):617–29. doi: 10.1007/s13412-025-01060-1 (PMC13428771; doi:10.1007/s13412-025-01060-1)
Supplement: Supplementary file 1 — Supplementary file1 [file 13412_2025_1060_MOESM1_ESM.docx]

**Supplemental Tables**

*Supplemental Table 1:* Global Moran’s *I* statistics for preterm birth (PTB), low birth weight (LBW), gestational diabetes mellitus (GDM), and pregnancy-induced hypertension (PIH).

| **Outcome** | **Global Moran’s *I*** |
| --- | --- |
| PTB | 0.297 *** |
| LBW | 0.443 *** |
| GDM | 0.284 *** |
| PIH | 0.268 *** |
| *** p < 0.001; ** p < 0.01; * p < 0.05. | |

*Supplemental Table 2:* Bivariate Moran’s *I* statistics for environmental stressors (e.g. heatwaves, toxicity, ICE Income, ICE Race) and preterm birth (PTB), low birth weight (LBW), gestational diabetes mellitus (GDM), and pregnancy-induced hypertension (PIH).

|  | **Heatwave** | **Toxicity** | **ICE Income** | **ICE Race** |
| --- | --- | --- | --- | --- |
| **PTB** | -0.031 | 0600. | -0.318 | -0.222 |
| **LBW** | 0.008 | 0.062 | -0.393 | -0.354 |
| **GDM** | -0.053 | -0.027 | -0.074 | 0.058 |
| **PIH** | -0.045 | -0.012 | -0.108 | 0.036 |

Supplemental Table 3. *Spatial Regression and Ordinary Least Square Regression Results for Low Birth Weight (LBW), Gestational Diabetes (GD), Pregnancy Induced Hypertension (PIH), and Preterm Birth (PTB), with structural racism defined using ICE Race: Black vs. Non-Hispanic white*

|  | GDM | | PIH | | LBW | | PTB | |
| --- | --- | --- | --- | --- | --- | --- | --- | --- |
|  | OLS | Spatial Lag | OLS | Spatial Lag | OLS | Spatial Lag | OLS | Spatial Lag |
| (Intercept) | 0.050*** | 0.015*** | 0.060*** | 0.020*** | 0.079*** | 0.052*** | 0.085*** | 0.055*** |
|  | (0.001) | (0.001) | (0.001) | (0.001) | (0.001) | (0.002) | (0.001) | (0.002) |
| Toxicity | −0.000 | −0.000 | −0.001* | −0.001* | 0 | −0.000 | 0.001 | 0 |
|  | (0.000) | (0.000) | (0.001) | (0.000) | (0.000) | (0.000) | (0.000) | (0.000) |
| Heatwaves | −0.002** | −0.000 | −0.002*** | −0.001 | −0.000 | −0.000 | −0.001* | −0.001 |
|  | (0.001) | (0.000) | (0.001) | (0.000) | (0.000) | (0.000) | (0.000) | (0.000) |
| Structural Racism | 0.008*** | 0.003** | 0.005*** | 0.001 | −0.023*** | −0.017*** | −0.013*** | −0.010*** |
|  | (0.001) | (0.001) | (0.001) | (0.001) | (0.001) | (0.001) | (0.001) | (0.001) |
| Income Inequality | 0.022*** | 0.012*** | 0.024*** | 0.011*** | 0.041*** | 0.032*** | 0.037*** | 0.028*** |
|  | (0.002) | (0.002) | (0.002) | (0.002) | (0.002) | (0.002) | (0.002) | (0.002) |
| Urban | Reference | | Reference | | Reference | | Reference | |
| Micropolitan | 0.002 | 0 | −0.003* | −0.003* | 0.001 | −0.001 | 0.001 | −0.000 |
|  | (0.001) | (0.001) | (0.001) | (0.001) | (0.001) | (0.001) | (0.001) | (0.001) |
| Rural | 0.011*** | 0.003 | 0.003 | −0.000 | −0.002 | −0.002 | 0.001 | −0.000 |
|  | (0.003) | (0.002) | (0.003) | (0.002) | (0.002) | (0.002) | (0.002) | (0.002) |
| Small Town | 0.005* | 0 | 0.002 | −0.001 | 0.001 | 0 | 0.002 | 0.001 |
|  | (0.002) | (0.002) | (0.002) | (0.002) | (0.002) | (0.002) | (0.002) | (0.002) |
| rho |  | 0.697*** |  | 0.663*** |  | 0.351*** |  | 0.360*** |
|  |  | -0.02 |  | -0.021 |  | -0.025 |  | -0.027 |
| Num.Obs. | 2165 | 2165 | 2165 | 2165 | 2165 | 2165 | 2165 | 2165 |
| R2 | 0.077 |  | 0.067 |  | 0.478 |  | 0.306 |  |
| R2 Adj. | 0.074 |  | 0.064 |  | 0.477 |  | 0.303 |  |
| AIC | −10183.7 | −11143.2 | −10067.9 | −10894.6 | −10964.9 | −11148.6 | −10664.8 | −10828.9 |
| *** *p* < 0.001; ** *p* < 0.01; * *p* < 0.05. | | | | | | | | |

Supplemental Table 4. *Spatial Regression and Ordinary Least Square Regression Results for Low Birth Weight (LBW), Gestational Diabetes (GD), Pregnancy Induced Hypertension (PIH), and Preterm Birth (PTB), with structural racism defined using ICE Race: Hispanic vs. Non-Hispanic white*

|  | GDM | | PIH | | LBW | | PTB | |
| --- | --- | --- | --- | --- | --- | --- | --- | --- |
|  | OLS | Spatial Lag | OLS | Spatial Lag | OLS | Spatial Lag | OLS | Spatial Lag |
| (Intercept) | 0.052*** | 0.016*** | 0.056*** | 0.018*** | 0.083*** | 0.050*** | 0.086*** | 0.054*** |
|  | (0.001) | (0.001) | (0.001) | (0.002) | (0.001) | (0.002) | (0.001) | (0.003) |
| Toxicity | −0.001 | −0.000 | −0.001* | −0.001* | 0.000 | 0.000 | 0.001 | 0.000 |
|  | (0.001) | (0.000) | (0.001) | (0.000) | (0.000) | (0.000) | (0.000) | (0.000) |
| Heatwaves | −0.002*** | −0.000 | −0.002** | −0.000 | 0.000 | 0.000 | −0.001 | −0.000 |
|  | (0.001) | (0.000) | (0.001) | (0.000) | (0.000) | (0.000) | (0.000) | (0.000) |
| Structural Racism | 0.004* | −0.001 | 0.012*** | 0.005** | −0.026*** | −0.019*** | −0.013*** | −0.010*** |
|  | (0.002) | (0.001) | (0.002) | (0.002) | (0.002) | (0.002) | (0.002) | (0.002) |
| Income Inequality | 0.017*** | 0.008*** | 0.026*** | 0.013*** | 0.048*** | 0.035*** | 0.041*** | 0.031*** |
|  | (0.002) | (0.002) | (0.002) | (0.002) | (0.002) | (0.002) | (0.002) | (0.002) |
| Urban | Reference | | Reference | | Reference | | Reference | |
| Micropolitan | 0.003+ | 0.001 | −0.004** | −0.003* | 0.000 | −0.001 | 0.001 | −0.001 |
|  | (0.001) | (0.001) | (0.001) | (0.001) | (0.001) | (0.001) | (0.001) | (0.001) |
| Rural | 0.012*** | 0.004+ | 0.002 | −0.001 | −0.003 | −0.003 | 0.000 | −0.001 |
|  | (0.003) | (0.002) | (0.003) | (0.002) | (0.002) | (0.002) | (0.002) | (0.002) |
| Small Town | 0.006* | 0.000 | 0.002 | −0.001 | 0.001 | 0.000 | 0.002 | 0.001 |
|  | (0.002) | (0.002) | (0.002) | (0.002) | (0.002) | (0.002) | (0.002) | (0.002) |
| rho |  | 0.704*** |  | 0.659*** |  | 0.412*** |  | 0.385*** |
|  |  | -0.019 |  | -0.021 |  | -0.025 |  | -0.027 |
| Num.Obs. | 2165 | 2165 | 2165 | 2165 | 2165 | 2165 | 2165 | 2165 |
| R2 | 0.063 |  | 0.076 |  | 0.43 |  | 0.284 |  |
| R2 Adj. | 0.06 |  | 0.073 |  | 0.428 |  | 0.282 |  |
| AIC | −10150.2 | −11135.4 | −10089.3 | −10902.4 | −10771.8 | −11032.5 | −10598.1 | −10788.2 |
| *** *p* < 0.001; ** *p* < 0.01; * *p* < 0.05. | | | | | | | | |
