# Supplementary figures and images for: Spatial analysis of environmental and socioeconomic factors impacting maternal and infant health outcomes in North Carolina
Source: J Environ Stud Sci. 2025 Nov 6;16(3):617–29. doi: 10.1007/s13412-025-01060-1 (PMC13428771; doi:10.1007/s13412-025-01060-1)

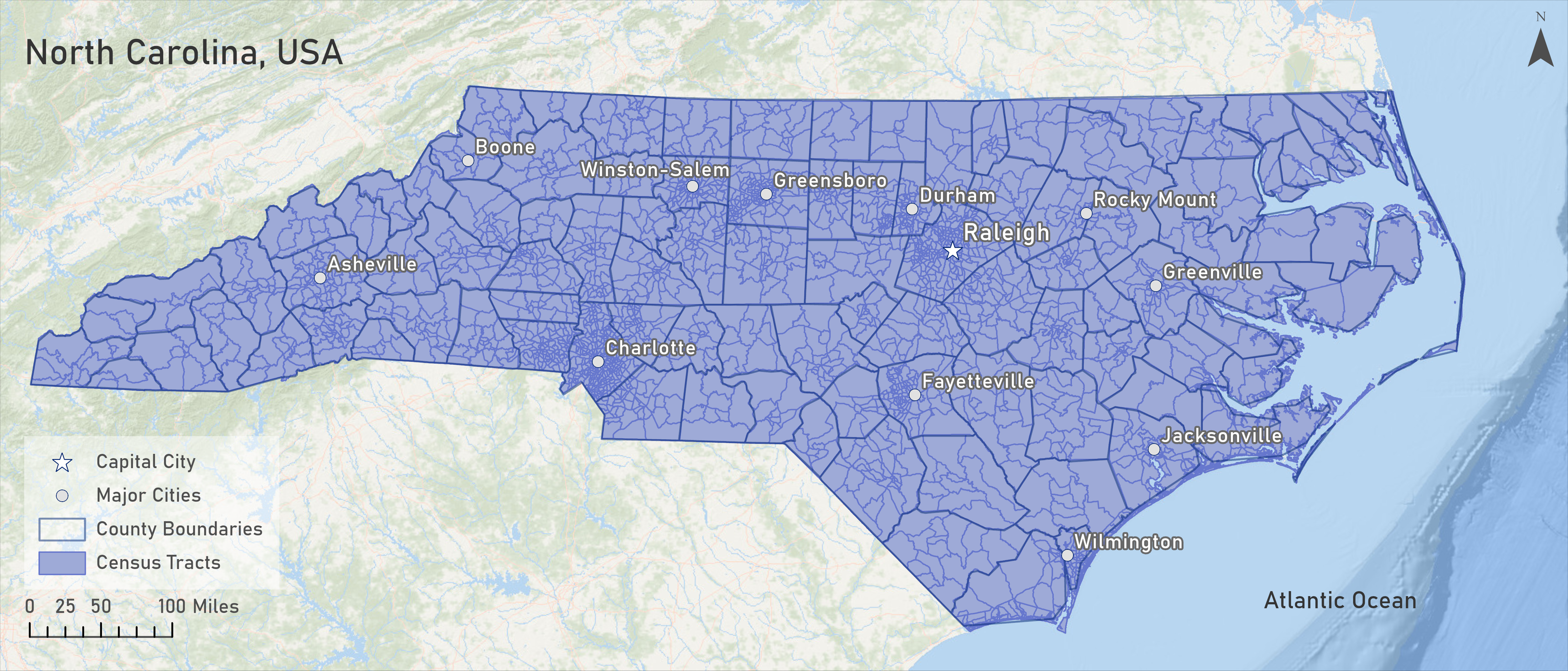

Supplement: Supplementary file 2 — Supplementary file2 [file 13412_2025_1060_MOESM2_ESM.png]

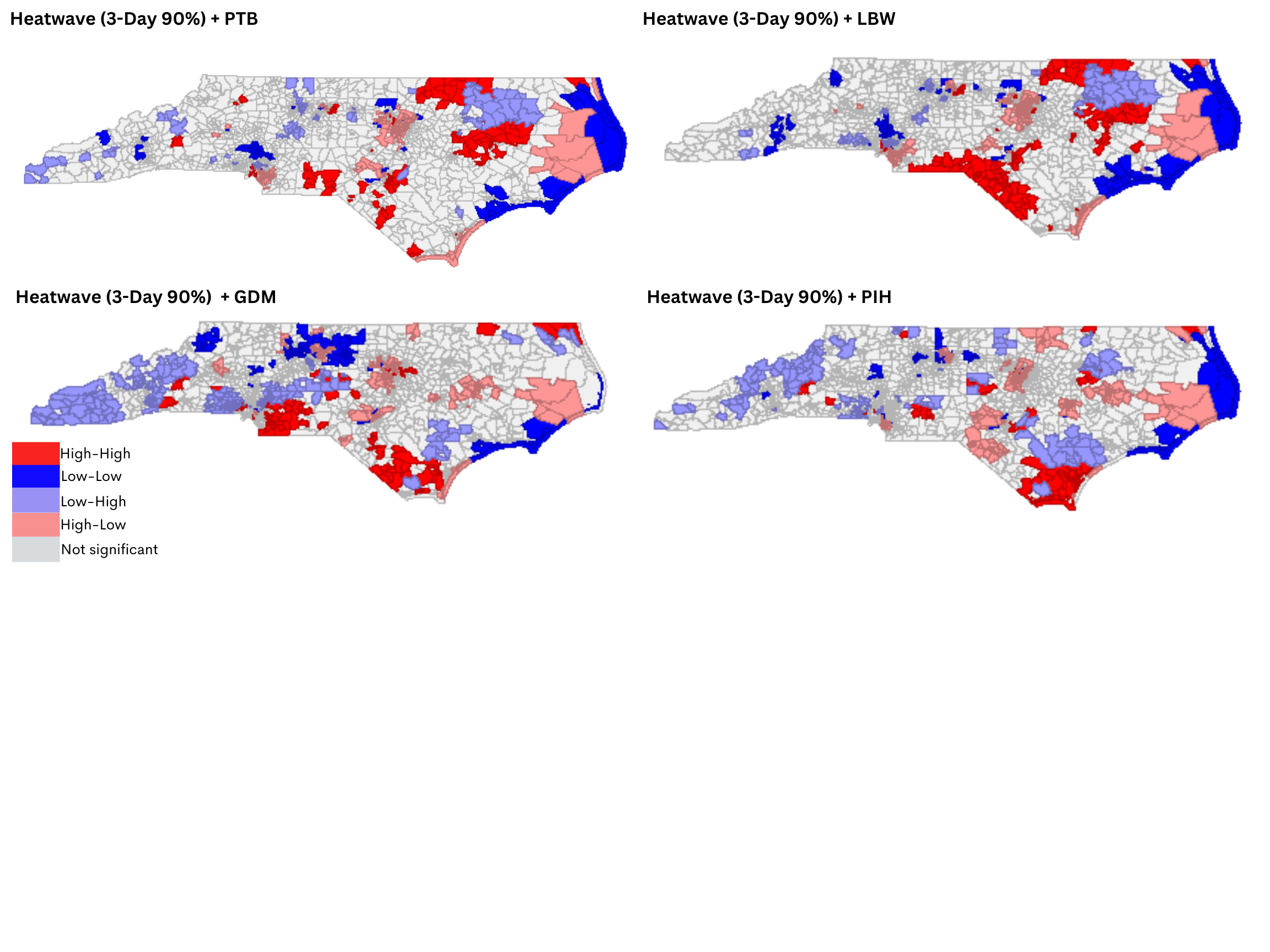

Supplement: Supplementary file 3 — Supplementary file3 [file 13412_2025_1060_MOESM3_ESM.png]

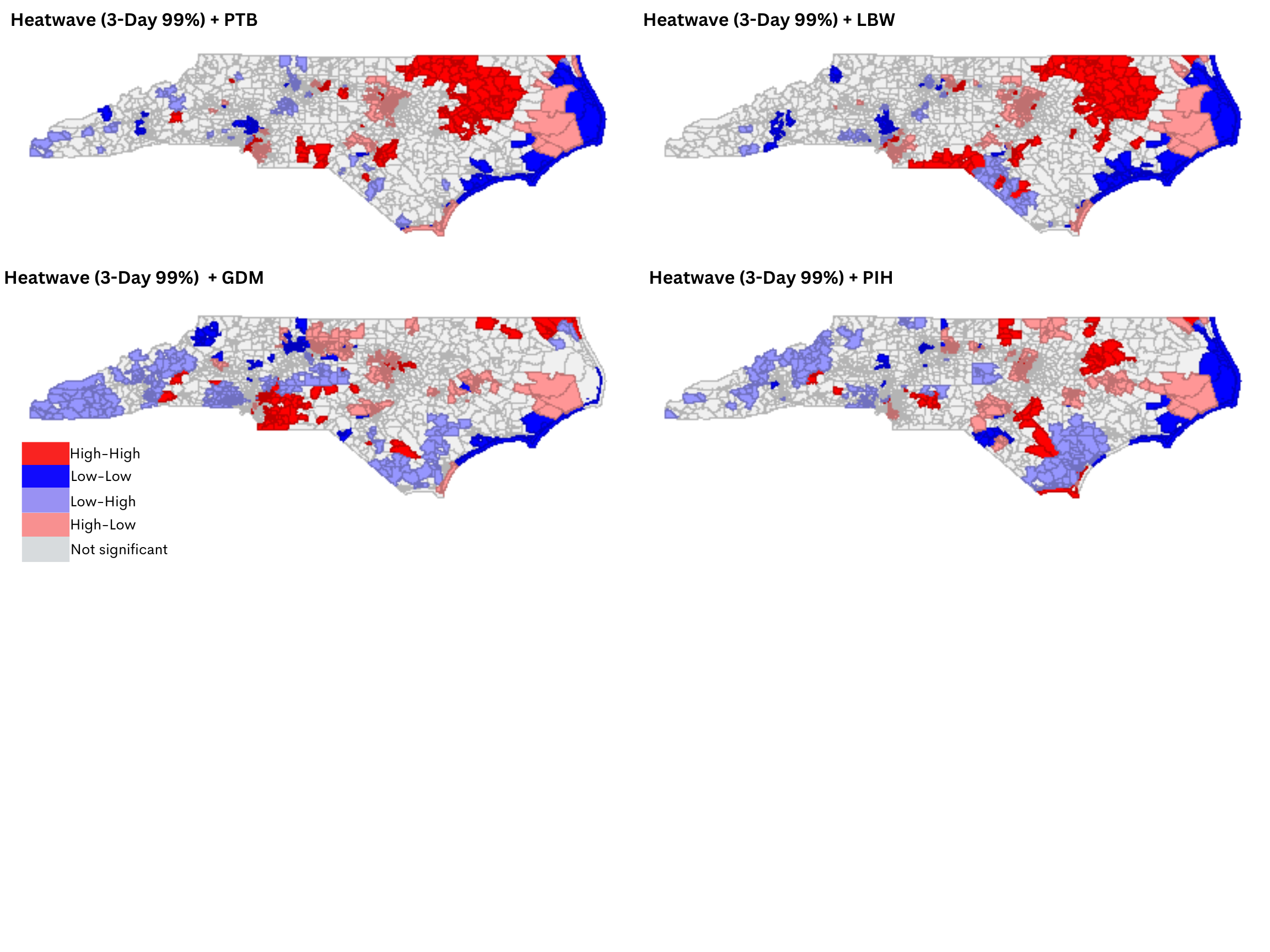

Supplement: Supplementary file 4 — Supplementary file4 [file 13412_2025_1060_MOESM4_ESM.png]
